# Supplementary material for: Optical Micromanipulations Based on Model Predictive Control of Thermoviscous Flows
Source: Small. 2025 Aug 13;21(38):e01039. doi: 10.1002/smll.202501039 (PMC12462561; doi:10.1002/smll.202501039)
Supplement: Supplementary file 1 — Supporting Information [file SMLL-21-e01039-s002.docx]

**Supporting Information**

**
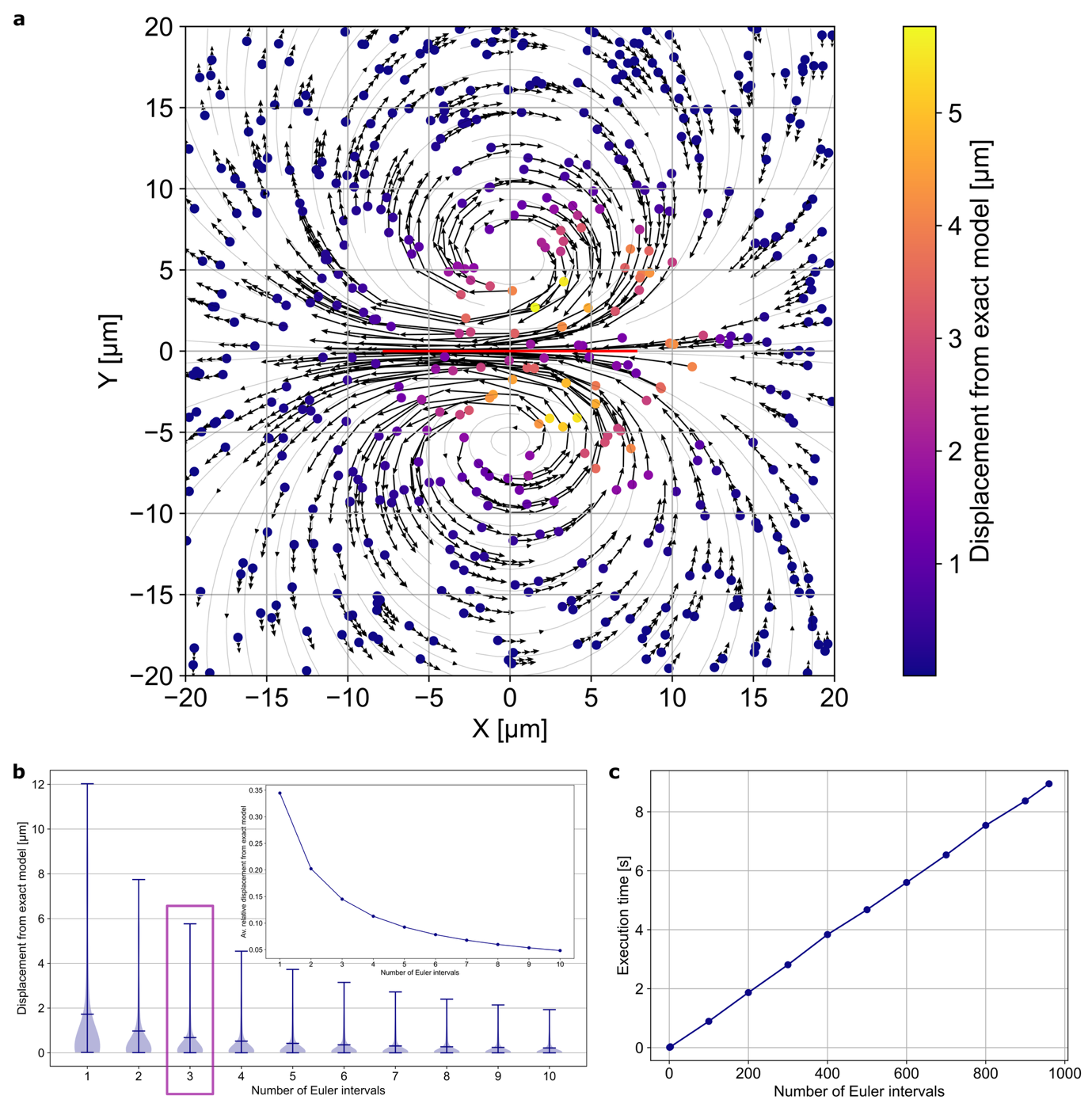
**

**Figure S1: Multi-step Euler approximation of the velocity field model used for predictive control in simulations and experiments.**

a) The panel depicts the approximated trajectories (black arrows) of 500 randomly distributed particles following the application of an IR laser scan path (red). These trajectories were modeled using a three-step Euler approximation of the analytical flow field model.[1] The background features the flow field streamlines, which were calculated according to the precise analytical model and are depicted in light grey. The particles are colored according to how much their final positions deviate from the exact model, depending strongly on their positions relative to the scan path.

b) The deviation of the Euler approximation from the exact model is shown to decrease with the number of Euler steps used. The inset graph shows the average relative displacement against the number of Euler steps used. The deviations depend on the absolute flow speed and the scan duration. The calculations in (a) and (b) were run for the same model parameters; the data of (a) forms the distribution for three Euler intervals in (b). For the approximation using three Euler steps (purple box), which we used in the experiment shown in Figure 3, the maximum expected deviation of the Euler approximation is 5.8 µm. As seen in (a) and (b), the deviation can be much smaller depending on a particle's position in the flow field.  As discussed in the Methods, the accuracy of the Euler approximation is one of many factors affecting the accuracy of the predictive step as a whole. The data emphasizes the robustness of the alignment method against inaccuracies in the model prediction.

c) The script execution time increases with the number of Euler intervals (shown here for 10 particles, run on a MacBook Pro, with Apple M3 Pro chip and 36 GB of RAM). To balance execution time and model accuracy, three Euler intervals were selected for predictive simulations in alignment experiments.

**
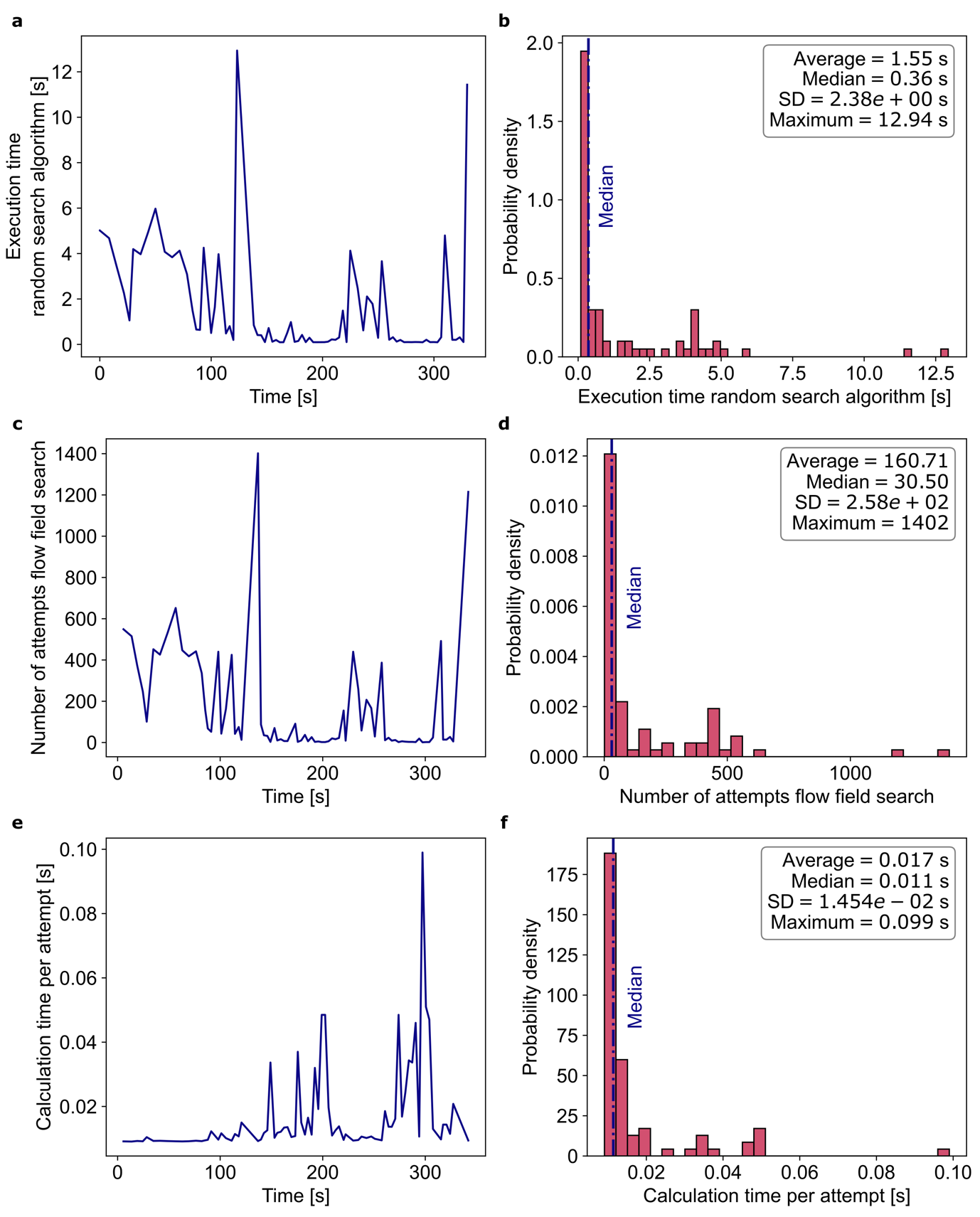
**

**Figure S2: Performance characterization of the random search algorithm.**

a) Time progression and b) histogram of execution time of the random search algorithm. The median time until a suitable flow field is found is 0.36 s.

c) Number of tested flow fields per execution step of the random search algorithm over time. d) The median number of tested flow fields is 30.5.

e) Time progression of calculation time per tested flow field and f) histogram.

Although there is some variability in the calculation time per operation (tested flow field), the variation in execution time of the random search algorithm is clearly caused by the variable number of attempts needed to find a suitable flow field.


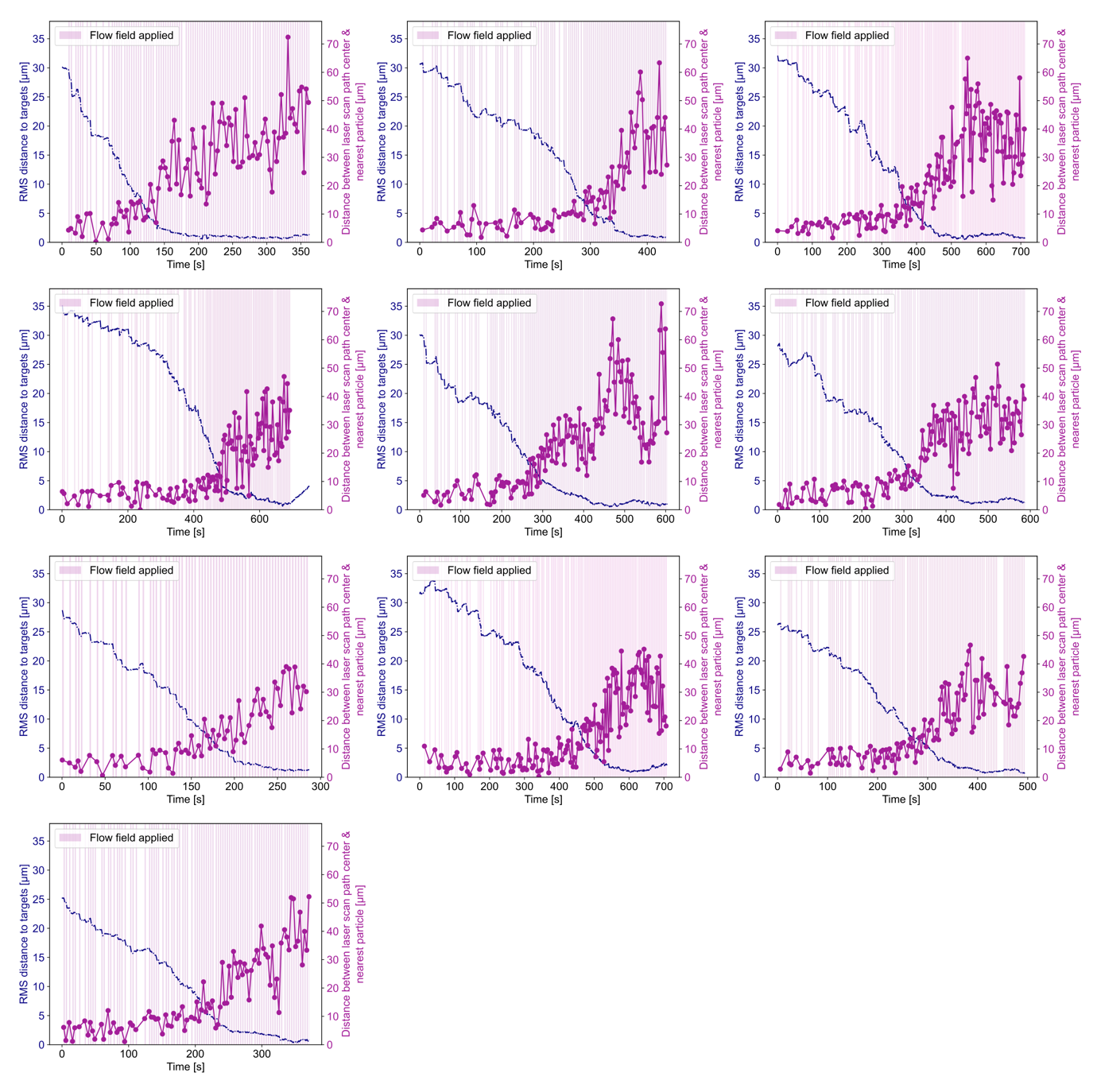


**Figure S3: Repetition of the alignment experiment of triangular arrangement (Figure 3) consistently shows an increase in the distance of the laser scan path to the nearest particle with alignment progression.**

The plots show consistent convergence of the objective function (RMS distance), but the time horizon for this convergence can vary due to the stochastic nature of the optimization approach.


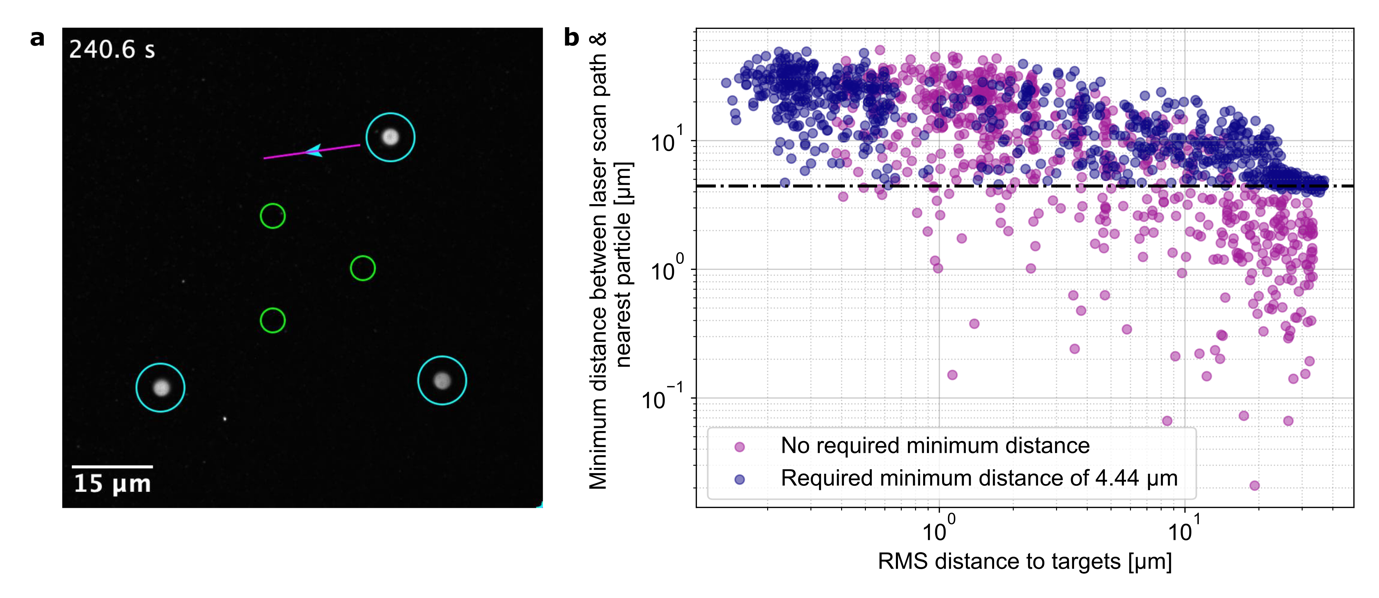


**Figure S4: The random search algorithm can be modified to respect the constraint of a minimum distance between the particles and the laser scan path.**

The random search algorithm was modified to maintain a minimum distance (illustrated by the cyan circles in **a**) between the infrared laser scan path and all particles. This was achieved by rejecting all randomly generated scan paths that violated this constraint, before modelling whether the scan path satisfied the objective function improvement condition. Alternatively, this constraint could have been incorporated in the objective function; however, this would likely have resulted in a substantial increase in the computational time of the random search algorithm.

A plot of the minimum distance between the scan path and the nearest particle (**b**) shows that the minimum distance requirement was largely fulfilled. The observed maximum deviation of 0.49 μm (11%) from the exact required value of 4.44 μm can be attributed to the displacement of particles due to diffusion during the period until the scan path has been calculated (see Fig. S3, Methods). Despite this deviation, the lowest measured distance of any particle to the scan path was 3.95 μm, which should still ensure reduced heating at the particle locations and prevent optical trapping effects of the particles.

It is important to distinguish the quantity plotted on the y-axis in this figure from the plot in Figure 3e. Here, the minimum distance between the particles and any point on the scan path is plotted. Conversely, in Figure 3e, the minimum distance between the particles and the center of the scan path is plotted. The data for the plots of both conditions are derived from a single experiment each.

It should be noted that certain parameters employed in this experiment differed from those utilized in the other experiments presented in this paper. In the following, the differing parameters are listed.

The required objective function improvement was 1% for the experiment without the minimum distance to the laser scan path (purple dots) and 0.5% for the experiment with the minimum distance to the laser scan path requirement (blue dots). In these experiments, the Lennard-Jones potential for interparticle repulsion was utilized, with a critical interparticle distance of  3.33 µm.

The camera field of view and the region from which the scan paths were sampled were the same size (88.8 µm square). A scan path length of 17.8 µm was utilized for both the modeled flow field and the executed scan path. The hardware of the optical setup control computer was upgraded (Intel(R) Xeon(R) w7-3555, 2.71 GHz, 128 GB RAM, 28 CPU cores), which enabled the execution of these experiments at a feedback sampling period of 0.17 s using 24 cores.

Additionally, the amplitude of the temperature spot (Equation 4) was modified to be constant throughout the scan path:

$A\left( t \right)=1$ . (5)

This configuration more accurately describes the temperature amplitude in our experimental setup, wherein the laser amplitude essentially remained constant throughout the scan period (i.e., essentially the “trapezoidal” amplitude function discussed by Liao et al.[1]). This resulted in a more than twofold increase (factor ca. 2.38) in the modeled velocities, while the shape of the velocity field in the near field underwent only a slight change. The far-field scaling remained unchanged. Consequently, due to the aforementioned changes in flow field speed, the empirical factor for the speed adjustment could be reduced to 1.37.

**Supplementary Videos:**

A note on color annotation: As in the main manuscript, green circles indicate the target locations and a magenta line represents the scan path, with a cyan arrow depicting the local direction of the flows.

- SV1: Three-dimensional rendering of exemplary data from stepwise deterministic simulations of the alignment of nine particles to first form a densely packed 3×3 grid-like target arrangement and then to separate to form a more loosely spaced version of the same target pattern. The video displays the full data from the simulation shown as snapshots in Figures 2a and 2b in the main manuscript. Particle motion is linearly interpolated between time steps.
- SV2: Three polystyrene particles are assembled into the shape of a triangle using model predictive control. The video shows the data analyzed in Figure 3 in the main manuscript.
- SV3: Assembly of nine polystyrene particles into a 3×3 grid using a random search algorithm as described in this paper. The video shows the data analyzed in Figure 4 in the main manuscript.
- SV4: An attempt to assemble a 3×3 grid from nine particles with the rule-based approach[2] shows the shortcomings of this approach compared to model predictive control.

**References**

[1] Liao, W., Erben, E., Kreysing, M., and Lauga, E. (2023) Theoretical model of confined thermoviscous flows for artificial cytoplasmic streaming. *Phys. Rev. Fluids*, **8** (3), 034202.

[2] Erben, E., Liao, W., Minopoli, A., Maghelli, N., Lauga, E., and Kreysing, M. (2024) Opto-fluidically multiplexed assembly and micro-robotics. *Light: Sci. Appl.*, **13** (1), 59.
